# Myeloid sarcoma of the nasal cavity in a 15-month-old child: A case report

**DOI:** 10.1097/MD.0000000000021119

**Published:** 2020-07-02

**Authors:** Ruowu Liu, Jintao Du, Limin Gao, Yafeng Liu, Shixi Liu

**Affiliations:** a Department of Otolaryngology-Head and Neck Surgery; b Department of Pathology, West China Hospital, Sichuan University, Chengdu, Sichuan, China.

**Keywords:** child, myeloid sarcoma, nasal cavity, neoplasm

## Abstract

**Introduction:**

Myeloid sarcoma (MS) is a rare tumor mass. It may occur at any extramedullary anatomic sites but is uncommon in the sinonasal location.MS commonly presents concurrently with acute myeloid leukemia (AML), but it may predate AML over several months or years, named isolated MS.

**Patient concerns:**

We report a case of a 15-month-old child who presented with mouth breathing, bilateral rhinorrhea, palpebral edema and proptosis. The routine blood tests were normal for the first few months. Computed tomography scan revealed neoplasm in nasal cavity.

**Diagnosis:**

The patient was definitely diagnosed with isolated MS in the nasal cavity through immunohistochemistry combined with clinical features and radiological investigations, and MS further progressed to AML which was confirmed by hematologist.

**Interventions:**

Endoscopic sinus surgery was performed to acquire specimens. After diagnosis, the patient was promptly treated with systemic chemotherapy.

**Outcomes:**

All symptoms gradually subsided and the mass of nasal cavity was invisible. No relapse occurred during follow-up.

**Conclusion:**

Sinonasal MS may be misdiagnosed and should be considered when symptoms persist and worsen. Prompt clinic examinations are essential for cases with suspected MS. Diagnosis of MS is dependent on the immunohistological investigations combined with clinical features, radiological investigations. Early diagnosis and systemic chemotherapy are vital for patients to achieve best prognosis.

## 1 Introduction

Myeloid sarcoma (MS) is a tumor mass consisting of myeloid blasts at an anatomic site other than the bone marrow.^[1]^ MS commonly originates in the lymph nodes, skin and gastrointestinal tract but rarely in the sinonasal location.^[2,3]^ MS may occur in isolation, or more commonly in patients with acute myeloid leukemia (AML), myeloproliferative neoplasm, myelodysplastic syndrome, or myeloproliferative neoplasm/myelodysplastic syndrome.^[4]^ The sinonasal area may be a predilection site of isolated MS.^[5]^ Although MS had been described in 1853, the number of the disease is still rare, resulted in the limitation of literatures that define clinical features, diagnosis, treatment and outcomes. Thus, case reports and retrospective series are essential for study and management of MS. Here, we describe a rare case of MS of the nasal cavity in a 15-month-old child. Informed consent was obtained from the patient's guardian for publication of this case report.

## 2 Case report

A 15-month-old male child presented to the local ear, nose, and throat outpatient department with a 2-month history of bilateral rhinorrhea and mouth breathing, and the patient was diagnosed as rhinitis. After drug therapy for 3 months in local hospital, the patient's symptoms did not improve but persisted and aggravated. During that time, routine blood tests were normal. As a refractory case, the patient was then admitted to our hospital.

**Figure 1:**
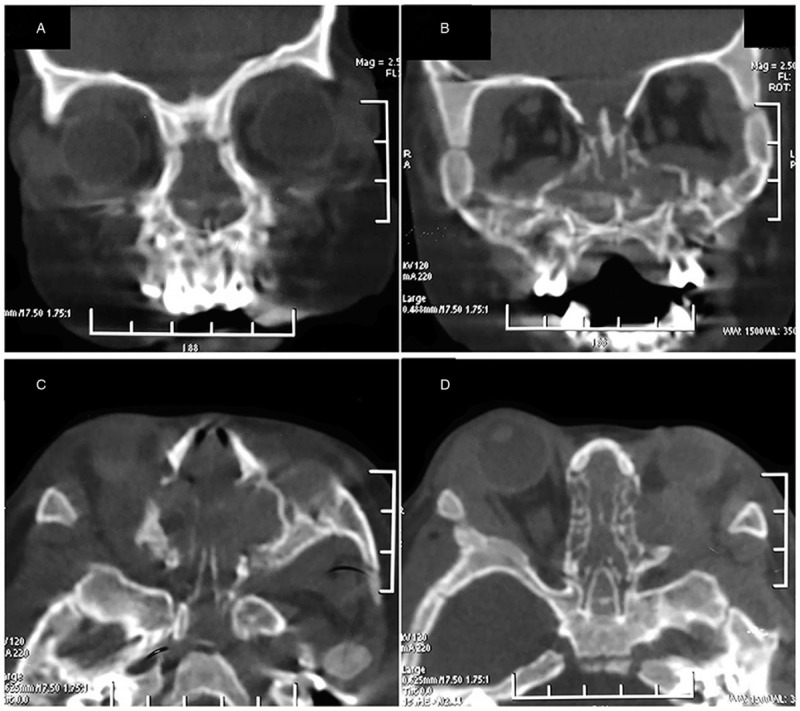
CT scan findings. Bilateral nasal cavity was full of tissue mass (A, B: Coronal view; C, D: Axial view). CT = computed tomograpghy.

**Figure 2:**
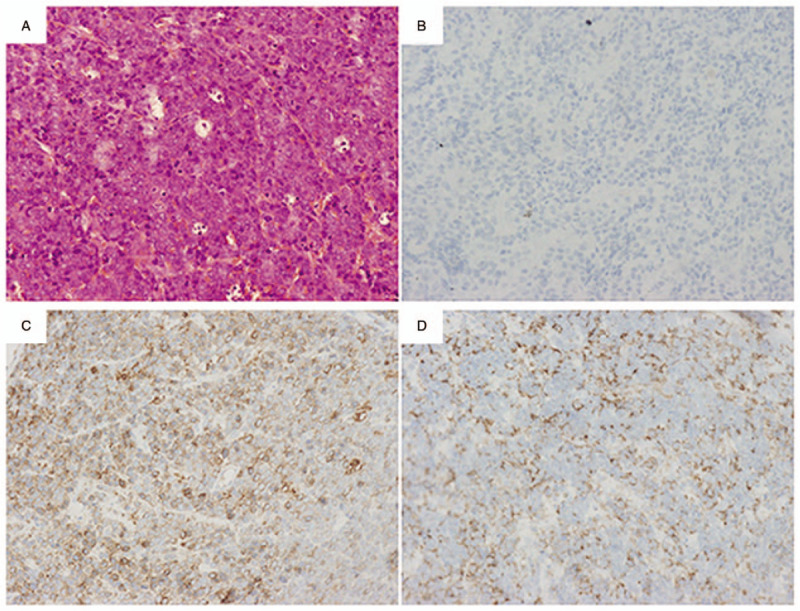
Pathological examination of nasal neoplasm. Histopathological examination showed the atypia of the specimen cells (A: H&E staining, × 200). Immunohistochemical staining showed that tumor cells are negatively immunostained for CD20 (B) and positively immunostained for myeloperoxidase (C) and CD99 (D).

In addition to rhinorrhea and mouth breathing, there existed bilateral palpebral edema and proptosis for this patient on admission. Nasal endoscopy verified neoplasm in bilateral nasal cavity. Computed tomography (CT) scan revealed that entire nasal cavity was occupied by tissue mass (Fig. 1). Hemogram showed increased leucocyte count (11.1 × 10^9^/L) including elevated myeloblast percentage (24.0%) and decreased neutrophil count (0.33 × 10^9^/L), and erythropenia (3.37 × 10^12^/L). Endoscopic sinus surgery under general anesthesia was performed to acquire specimens. A diagnosis of MS was made through postoperative pathology. Immunohistological analysis demonstrated that the mass was mainly infiltrated with atypical cells which were positive for myeloperoxidase, CD99, CD45 and Ki-67, but not for CD3, CD10, CD20, CD30, CD34, and CD117 (Fig. 2).

Postoperative hemogram showed remarkable leukocytosis (36.0 × 10^9^/L), as well as elevated myeloblast percentage (57%). The patient was promptly transferred to the hematology department, and further AML was confirmed by hematologist. It was 5 months since MS occurred in this patient. A week after the surgery, the patient then received conventional chemotherapy containing 5 phases to treat MS and AML. The first and second phases were induction chemotherapy consisted of daunorubicin for 3 days, cytarabine for 7 days and etoposide for 5 days. The remaining phases were intensification chemotherapy: intensification I-cytarabine for 3 days and mitoxantrone for 2 days; intensification II-cytarabine for 3 days and homoharringtonine for 7 days; and intensification III- cytarabine for 3 days.

**Figure 3:**
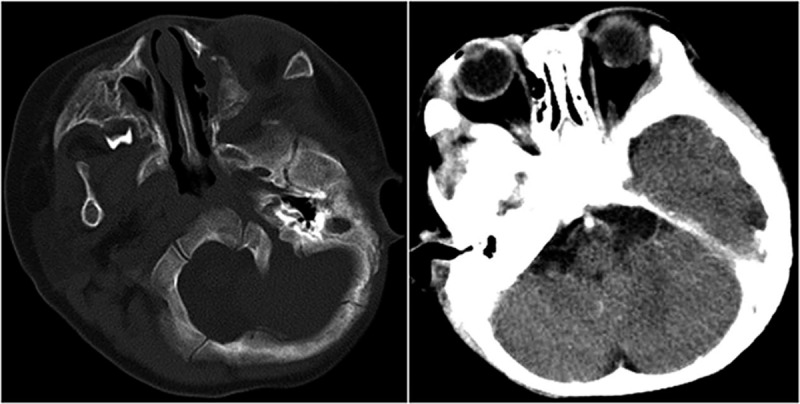
CT scans revealed that the tissue mass in nasal cavity was invisible. CT = computed tomograpghy.

Three weeks after the initiation of chemotherapy, symptoms of mouth breathing, rhinorrhea, palpebral edema and proptosis gradually subsided, and CT scan revealed that the mass of nasal cavity was invisible (Fig. 3). Five months later, at the completion of chemotherapy, the patient achieved complete remission eventually. No relapse occurred during 24 months’ follow-up.

## 3 Discussion

Although MS represents an extramedullary proliferation and may occur at nearly any anatomic site, MS involvement in the sinonasal area is rare. Suzuki et al^[5]^ summarized 10 sinonasal MS cases that had been reported between 2000 and 2018, including 9 adults and 1 child. Holsinger et al^[6]^ found 2 pediatric sinonasal MS cases recorded between 1955 and 1999 at university-based, tertiary care referral centers in Houston.

MS commonly presents concurrently with AML, but it may predate AML over several months or years, named isolated MS, which means it can appear before clinical signs of hematological disease.^[4,7]^ Klco et al^[3]^ deemed that no matter blood film or bone marrow biopsy is abnormal or not, the existence of MS is sufficient to establish a clinical diagnosis of AML, since bone marrow disease will develop in nearly all patients who originally presented with isolated MS, with a mean interval of 10 months .Based on the definition of isolated MS, we infer that this patient initially presented with isolated MS, and AML did not occur until 5 months later.

MS is an uncommon tumor mass affecting 2.9% to 9.1% of patients in AML, and isolated MS is a very rare entity with an incidence of 2 cases per million adults.^[4]^ However, the sinonasal area may be a predilection site of isolated MS occupied 20% of sinonasal MS cases.^[5]^ MS has a wide age distribution and pediatric population is a preference age group.^[3]^ Nevertheless, some retrospective series suggested the main age range of onset of MS is connected with tumor site to some extent.^[8,9]^ Sinonasal MS is almost seen in adult population but orbital MS is more common in pediatric population.^[5,10]^ Interestingly, their anatomical locations are relatively close. Accordingly, sinonasal MS in child is exceedingly rare. To our knowledge, this report describes the youngest case of sinonasal MS.

Due to its rarity, the diagnosis of MS, especially isolated MS, could be highly challenging. A diagnosis of MS is based on a combination of clinical features, radiological investigations, and immunohistochemistry.^[4]^ Clinical presentation is depended on size and localization. Sinonasal MS generally causes non-specific symptoms such as nasal obstruction, headache and tumor mass effects including facial swelling, proptosis and visual disturbance.^[5]^ Primally, our patient was misdiagnosed as rhinitis on account of limited non-specific symptoms and normal blood film, and then because of aggravated nasal symptoms and additional palpebral edema and proptosis with suspicion of malignant neoplasm, nasal endoscopy and CT scan were performed. CT scan enable evaluation of size and location of the tumor and could distinguish tumor from other lesions, such as hematomas or abscess, but definite diagnosis of MS is dependent on the biopsy of the tumor and immunohistochemical staining.^[11]^ For isolated MS, normal blood film or bone marrow biopsy is essential which, however, also resulted in a misdiagnosis rate of 25% to 47% of isolated MS.^[4]^ Therefore, diagnosis of isolated MS requires a high index of suspicion as well as significant clinical acumen.^[4]^

The treatment of MS include systemic chemotherapy, local therapy involving either surgery or radiotherapy or a combination, and hematopoietic stem cell transplantation (HSCT).^[11]^ Local therapy alone neither appears to delay the transformation from MS to AML or improve the prognosis, which is not recommended.^[4]^ Two patients with sinonasal MS who received local therapy alone were dead in short time.^[12,13]^ The role of HSCT was highlighted since it improves the overall survival of patients with MS.^[11]^ Suzuki et al reviewed 2 sinonasal MS patients underwent HSCT, and both achieved satisfactory short-term survival.^[5]^ Systemic chemotherapy should be commenced as soon as possible in all cases including isolated MS which will transform to AML eventually.^[4]^ Chemotherapy can increase overall survival and delay or even prevent progression to AML in isolated MS.^[4]^ Because MS is a systemic disease and respond to systemic treatment, chemotherapy could remit symptoms which was caused by extramedullary lesion. Therefore, early diagnosis and chemotherapy are crucial to the prognosis of MS, especially isolated MS. Through chemotherapy, our patient achieved clinical and hematologic remission and have the longest survival compared to other adult patients with sinonasal MS.^[5]^ Originally presented with isolated MS and younger age may be positive prognostic factors. Several series demonstrated outcomes in children with isolated MS are better than those with MS concurrent or following AML.^[4,11]^ Furthermore, Avni et al^[14]^ showed that age less than 47.5 years was associated with a lower risk of death.

In conclusion, we report the youngest case of sinonasal MS who achieved the longest survival among sinonasal MS patients. The patient initially presented with isolated MS which did not progress to AML until 5 months later. MS, especially isolated MS, is highly misdiagnosed. Prompt clinic examinations are essential for cases with suspected MS. Definite diagnosis of MS is dependent on the immunohistological investigations combined with clinical features, radiological investigations. Early diagnosis and systemic chemotherapy are vital for patients to achieve best prognosis.

## Author contributions

**Conceptualization:** Jintao Du, Ruowu Liu.

**Methodology:** Yafeng Liu.

**Resources:** Limin Gao, Yafeng Liu.

**Supervision:** Shixi Liu.

**Writing – original draft:** Ruowu Liu.

**Writing – review & editing:** Jintao Du.

## References

[1] Wilson CS Medeiros LJ Extramedullary Manifestations of Myeloid Neoplasms. Am J Clin Pathol 2015;144:219. 26185307 10.1309/AJCPO58YWIBUBESX

[2] Prades JM Alaani A Mosnier JF Granulocytic sarcoma of the nasal cavity. Rhinology 2002;40:159–61. 12357718

[3] Klco JM Welch JS Nguyen TT State of the art in myeloid sarcoma. Int J Lab Hematol 2011;33:555–65. 21883967 10.1111/j.1751-553X.2011.01361.x

[4] Almond LM Charalampakis M Ford SJ Myeloid sarcoma: presentation, diagnosis, and treatment. Clin Lymphoma Myeloma Leuk 2017;17:263–7. 28342811 10.1016/j.clml.2017.02.027

[5] Suzuki J Harazaki Y Morita S Myeloid sarcoma of the paranasal sinuses in a patient with acute myeloid leukemia. Tohoku J Exp Med 2018;246:141–6. 30369515 10.1620/tjem.246.141

[6] Holsinger FC Hafemeister AC Hicks MJ Differential diagnosis of pediatric tumors of the nasal cavity and paranasal sinuses: a 45-year multi-institutional review. Ear Nose Throat J 2010;89:534–40. 21086277

[7] Campidelli C Agostinelli C Stitson R Myeloid sarcoma: extramedullary manifestation of myeloid disorders. Am J Clin Pathol 2009;132:426–37. 19687319 10.1309/AJCP1ZA7HYZKAZHS

[8] Semra P Suzan Z Melek E Granulocytic sarcoma: 32 cases and review of the literature. Leuk Lymphoma 2006;47:2527–41. 17169797 10.1080/10428190600967196

[9] Pileri SA Ascani S Cox MC Myeloid sarcoma: clinico-pathologic, phenotypic and cytogenetic analysis of 92 adult patients. Leukemia 2007;21:340–50. 17170724 10.1038/sj.leu.2404491

[10] Qian X Gigantelli JW Abromowitch M Myeloid sarcoma in the orbit. J Pediatr Ophthalmol Strabismus 2016;53:e64. 27977030 10.3928/01913913-20161102-01

[11] Samborska M Derwich K Skalska-Sadowska J Myeloid sarcoma in children - diagnostic and therapeutic difficulties. Contemporary oncology (Poznan, Poland) 2016;20:444–8. 10.5114/wo.2016.65602 PMC5320455 28239280

[12] Ferri E Minotto C Ianniello F Maxillo-ethmoidal chloroma in acute myeloid leukaemia: case report. Acta Otorhinolaryngol Ital 2005;25:195–9. 16450777 PMC2639872

[13] Kuo CL Yu YB Li WY Unusual coexistence of sinonasal myeloid sarcoma and acute fulminant invasive fungal sinusitis: a diagnostic dilemma. J Laryngol Otol 2013;127:415–8. 23448530 10.1017/S0022215113000285

[14] Avni B Rund D Levin M Clinical implications of acute myeloid leukemia presenting as myeloid sarcoma. Hematol Oncol 2012;30:34–40. 21638303 10.1002/hon.994

